# Patterns of perceived barriers to medical care in older adults: a latent class analysis

**DOI:** 10.1186/1472-6963-11-181

**Published:** 2011-08-03

**Authors:** Joshua M Thorpe, Carolyn T Thorpe, Korey A Kennelty, Nancy Pandhi

**Affiliations:** 1 Division of Social & Administrative Sciences, University of Wisconsin-Madison School of Pharmacy. 777 Highland Ave, Madison, WI, 53719, USA; 2 Sonderegger Research Center, University of Wisconsin-Madison School of Pharmacy. 777 Highland Ave, Madison, WI, 53719, USA; 3 Health Innovation Program, Department of Population Health Sciences, University of Wisconsin School of Medicine and Public Health. 750 Highland Ave, Madison, WI, 53719, USA; 4 Department of Family Medicine, University of Wisconsin School of Medicine and Public Health. 750 Highland Ave, Madison, WI, 53719, USA

## Abstract

**Background:**

This study examined multiple dimensions of healthcare access in order to develop a typology of perceived barriers to healthcare access in community-dwelling elderly. Secondary aims were to define distinct classes of older adults with similar perceived healthcare access barriers and to examine predictors of class membership to identify risk factors for poor healthcare access.

**Methods:**

A sample of 5,465 community-dwelling elderly was drawn from the 2004 wave of the Wisconsin Longitudinal Study. Perceived barriers to healthcare access were measured using items from the Group Health Association of America Consumer Satisfaction Survey. We used latent class analysis to assess the constellation of items measuring perceived barriers in access and multinomial logistic regression to estimate how risk factors affected the probability of membership in the latent barrier classes.

**Results:**

Latent class analysis identified four classes of older adults. Class 1 (75% of sample) consisted of individuals with an overall low level of risk for perceived access problems (No Barriers). Class 2 (5%) perceived problems with the availability/accessibility of healthcare providers such as specialists or mental health providers (Availability/Accessibility Barriers). Class 3 (18%) perceived problems with how well their providers' operations arise organized to accommodate their needs and preferences (Accommodation Barriers). Class 4 (2%) perceived problems with all dimension of access (Severe Barriers). Results also revealed that healthcare affordability is a problem shared by members of all three barrier groups, suggesting that older adults with perceived barriers tend to face multiple, co-occurring problems. Compared to those classified into the No Barriers group, those in the Severe Barrier class were more likely to live in a rural county, have no health insurance, have depressive symptomatology, and speech limitations. Those classified into the Availability/Accessibility Barriers group were more likely to live in rural and micropolitan counties, have depressive symptomatology, more chronic conditions, and hearing limitations. Those in the Accommodation group were more likely to have depressive symptomatology and cognitive limitations.

**Conclusions:**

The current study identified a typology of perceived barriers in healthcare access in older adults. The identified risk factors for membership in perceived barrier classes could potentially assist healthcare organizations and providers with targeting polices and interventions designed to improve access in their most vulnerable older adult populations, particularly those in rural areas, with functional disabilities, or in poor mental health.

## Background

The medical care needs of older adults are often considerable. Approximately 80% of older adults require ongoing care for at least one chronic condition, 50% have multiple chronic conditions, and 60% are managing three or more prescription medications [1]. Even in the absence of chronic illness, older adults need to access medical care for acute conditions as they arise, as well as for extensive preventive care services recommended by evidence-based guidelines (e.g., annual influenza vaccination; screening for hypertension, hypercholesterolemia, and many cancers) [2,3]. Access to a range of health services, therefore, is critically important for preventing new illnesses, adapting therapies to changing needs, potentially reducing acute care costs, and ultimately for maintaining the health and well-being of our aging population [4-6]. As this vulnerable population is expected to double by the year 2030, efforts to identify and eliminate disparities in access for older adults are among the most pressing health care issues for the 21st century [7]. One current proposed solution for reducing barriers in access is through the patient-centered medical home - a health care delivery model whereby a personal physician leads a team of individuals to coordinate accessible care for a patient across health care settings [8]. Older and vulnerable adults are known to have a strong preference for continuity with an individual physician [9], and initial studies of medical home have shown encouraging results in regards to reducing disparities in access to care [10] and improving the quality of care [11].

Despite the importance of identifying and eliminating barriers in access, there is little consistency in the approaches used to measure its presence. The majority of research on access to care in older adults has focused on actual use of healthcare services (e.g., doctor visits, receipt of preventive care) and its determinants (e.g., financial barriers, insurance status, rurality, racial/ethnic minority status) [12-18]. While the study of disparities in use of healthcare services can be helpful for identifying potential inequities across populations, there are a number of limitations to this approach. First, equating *use *of health services (or failure to use services) to *access *to health services can be misleading and may mask significant obstacles faced by patients when seeking necessary medical care. For instance, even those that use healthcare services may have had to overcome substantial barriers to do so, and conversely, lack of use does not necessarily mean patients had poor access. Asking people to report their perceptions of access to care rather than relying on actual patterns of service use, therefore, may be a preferred method of more directly measuring their true access. Second, access is a complex construct that is comprised of multiple, distinct dimensions (e.g., affordability of services, availability of providers, accommodating office hours, etc.) [19]. Intervention and policy solutions designed to improve access to care in older adults may, therefore, vary greatly depending on the dimension(s) in which perceived barriers are reported by patients. Finally, barriers to care along the distinct access dimensions may, more realistically, co-occur and interact in ways that differentially impact older adults' ability to obtain needed services, and may require different intervention approaches. Research that reveals co-occurring barriers across the range of access dimensions may yield additional insights into the constellation of barriers that older adults may simultaneously experience.

One promising approach to the study of access to care that acknowledges both the multidimensionality of access and the potential for co-occurring barriers is to conceptualize access as a latent categorical variable in order to group individuals reporting similar types of barriers to care [20,21]. This typological approach to the concept of access is also known as a "person-centered" approach, and examples of such methods include latent class analysis [20], cluster analysis [22], and growth mixture models [23]. By using a person-centered method to delineate subgroups of individuals who perceive similar types of access barriers, it may be possible to tailor intervention and policy efforts to more accurately reflect the issues faced by different subgroups of older adults in obtaining timely and effective health care.

The purpose of this study, therefore, was to use a person-centered approach (i.e., latent class analysis) to simultaneously examine multiple dimensions of access in order to develop a typology of perceived healthcare access barriers in community-dwelling elderly. The specific study aims were to: (1) use latent class analysis to classify older adults with similar perceived access barriers; (2) provide evidence of latent class concurrent validity by examining association between class membership and patterns of health service utilization and unmet healthcare needs; and (3) explore factors predicting class membership to identify risk factors (and thus, possible points of intervention) for poor access.

## Methods

### Conceptual Model

**Figure 1:**
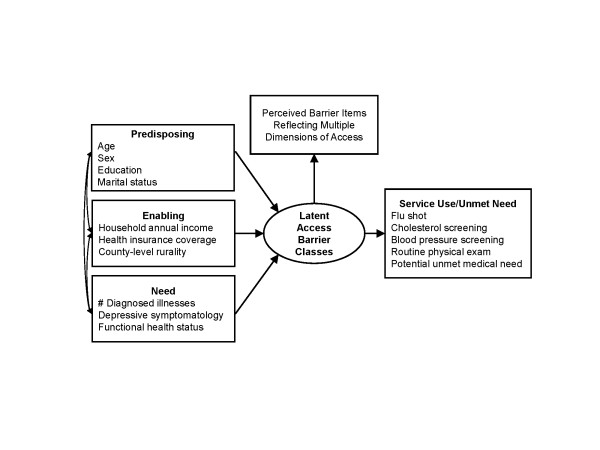
**Model of the relationships among Andersen SBM variables, latent access barrier classes, and unmet need**. Andersen SBM variables were grouped as predisposing, enabling and need variables. Unmet need was measured as both self-reported unmet need and clinical preventive care use.

Figure 1 shows our conceptual model of the causal process leading from upstream determinants of health care utilization to perceptions of access to care, and ultimately to actual health service utilization. A modified version of Andersen's sociobehavioral model (SBM) of health service use [24] serves as the basis for our selection of upstream factors that may shape respondent's perceptions about access to health care. The SBM includes three domains of these upstream factors: predisposing, enabling, and medical need. Predisposing variables are intrapersonal factors that affect one's propensity for using health services (e.g., demographics). Enabling variables are factors that either facilitate or impede access to health services (e.g., health insurance). Medical need refers to the individual's illnesses or impairments that necessitate health service use (e.g., chronic illnesses). These upstream predisposing, enabling, and need variables are hypothesized to determine one's latent barrier class membership. Although not directly observable, latent class membership is inferred from patterns of perceived barriers in health care access.

We organize perceived access items into their corresponding dimensions of access described by Penchansky: availability/accessibility, acceptability, accommodation, and affordability [19]. Availability and accessibility are related constructs pertaining to the adequacy of supply of healthcare providers and location of providers relative to patients. Acceptability is defined as the interaction between patient and provider attitudes and preferences about what constitutes acceptable personal and treatment practices, with a focus on patient trust in the current study. Accommodation refers to how well the providers' operations are organized to accommodate patients' constraints and preferences. Affordability refers to the relationship of prices of services to patient income, insurance, and overall ability to pay. Finally, the conceptual model suggests that latent barrier class membership shapes respondent health service utilization behaviors.

### Sample

The sample was drawn from the 2004 wave of the Wisconsin Longitudinal Study (WLS), a longitudinal study of a random sample of 10,317 graduates from Wisconsin high schools in 1957. The study design and history of the WLS have been described in detail elsewhere [25]. All WLS data, documentation, and questionnaires are publicly available http://www.ssc.wisc.edu/wlsresearch. The WLS has also been stripped of personal identifiers. Therefore, the University of Wisconsin - Madison Institutional Review Board has determined that secondary analyses of the publicly available WLS data do not rise to the level of human subjects research and, therefore, do not require IRB review.

In 2004, 8,578 surviving WLS graduates were invited to participate, and 6,279 (73%) completed both the phone and mail interviews. We further excluded 814 respondents who did not complete the prior WLS mail survey that was fielded in 1992 (n = 5,465) in order to facilitate sensitivity analyses incorporating data from this prior round of data collection.

### Measures

#### Perceived Access

The 2003/2004 WLS included 15 items pertaining to perceived access to health care. Twelve items originated from the access to care subscale of the Group Health Association of America Consumer Satisfaction Survey (CSS) [26]. Factor analysis from previous work on the CSS revealed four distinct dimensions [27], with item factor loadings corresponding to four of the access dimensions described by Penchansky and Thomas: availability/accessibility, accommodation, affordability, and acceptability. The CSS items loading on the availability/accessibility dimensions of access were as follows: (item 1) access to medical care in an emergency [emergency care], (item 2) access to hospital care [hospital care], (item 3) services available for getting prescriptions filled [prescription services], (item 4) access to specialty care if you need it [specialty care], (item 5) access to mental health care [mental health care], (item 6) convenience of the location of the doctor's office [convenience of office location]. CSS items loading on the accommodation dimension of access were as follows: (item 7) availability of medical information or advice by phone [advice by phone], (item 8) arrangements for making appointments for medical care by phone [phone appointments], (item 9) length of time you wait between making an appointment for routine care and the day of your visit [wait for appointment], (item 10) length of time spent waiting at the office to see the doctor [time in waiting room], (item 11) amount of time you have with doctors and staff during a visit [time with doctor]. The CSS item representing the affordability dimension of access was item 12, ability to afford health care costs [out-of-pocket costs]. In addition to CSS items, the WLS evaluated respondents' perceptions of interpersonal aspects of health care providers, representing the acceptability dimension of access, by asking respondents the extent to which they agreed that the doctor: (item 13) is totally honest about all treatment options available [doctor is honest]; (item 14) always pays complete attention to what the patient is saying [doctor pays attention]; (item 15) would share embarrassing information about you [doctor shares embarrassing information]. The original response categories were excellent, very good, good, fair, or poor. Responses of "fair" or "poor" were considered indicative of perceiving an access barrier; thus, response categories were collapsed as follows: 1 = fair/poor, 0 = good, very good, or excellent.

#### Covariates (SBM variables)

Predisposing variables are intrapersonal factors that affect one's propensity for using health services (e.g., demographics), and in this study included age (years), sex (0 = female, 1 = male), marital status (0 = unmarried, 1 = married), and years of formal education.

Enabling variables are factors that either facilitate or impede access to health services. Individual-level enabling variables included total household income, health insurance coverage (0 = any private insurance, 1 = public insurance only, 2 = no insurance), and county-level rurality defined using 2003 county-level Urban Influence Codes (UIC) [28], collapsed into categories of large and small metropolitan, micropolitan, and rural (non-core).

Medical need refers to the individual's illnesses or impairments that necessitate health service use, and in this study included number of diagnosed conditions, depressive symptomatology, and functional health status. In the WLS, diagnoses of 21 common chronic conditions were assessed via self-report by asking respondents for each of the 21 conditions, "*Has a doctor told you that you have*...". We created one variable representing the count of the total number of diagnosed conditions. Depressive symptoms were measured using the Center for Epidemiologic Studies Depression Scale (CES-D), a 20-item self-report scale designed to identify depression in the general population and community-dwelling older adults [29]. Scores range from 0 to 60, with higher scores indicating greater depressive symptoms (Cronbach's alpha = 0.88). We analyzed total CES-D scores as a dichotomous variable using scores greater than or equal to 16 to indicate depressive symptomatology [30]. Functional health limitations were assessed via the Health Utilities Index Mark III (HUI-III) [31]. We created dichotomous variables representing the presence of any reported limitation with regards to ambulation, dexterity, cognition, pain, speech, and hearing.

#### Latent Class Validation Items (Unmet need or delayed care)

To assess the concurrent validity of latent classes of perceived access barriers, we examined the association between latent classes and the following: (1) receipt of the flu shot in past 12 months (0 = no, 1 = yes), (2) screened for high cholesterol in past 12 months (0 = no, 1 = yes), (3) screened for high blood pressure in past 12 months (0 = no, 1 = yes); (4) received routine physical examination in past 12 months (0 = no, 1 = yes); and (5) any unmet medical need in past 12 months (0 = no, 1 = yes). Respondents were coded as having an unmet medical need or delayed care in the past 12 months if they reported difficulty or delay in seeking any type of medical care for the following reasons: could not afford, too far away, took too long to get an appointment, couldn't get through on the telephone to make an appointment, or couldn't get there when the doctor's office was open. All measures were based on respondent self-report. We hypothesized that respondents in access barrier groups would be less likely to receive recommended preventive care, and more likely to experience an unmet medical need or delayed care.

Some respondents had missing data on one or more variables. To reclaim cases with missing data, and those with less than 10% missing, we used conditional mean imputation to generate a single complete data set [32].

To analyze aim 1, we used latent class analysis (LCA) to simultaneously examine the full constellation of items measuring perceived barriers in health care access [33,34]. LCA, a probabilistic clustering approach, assumes that a set of substantively related categorical response variables reflect meaningful latent, discrete characteristics of individuals. In other words, we assume that a small number of distinct respondent subtypes (i.e., latent classes) exist among the measures representing attributes of healthcare access. With LCA, we obtain the probabilities of belonging to each of the estimated classes as well as the conditional probability of reporting a specific type of barrier given membership in a latent class. The result is a set of clusters in which older adults facing similar perceived barriers are grouped together. Whereas standard cluster analysis methods use somewhat arbitrary cluster criterion, LCA is a statistical model-based approach that allows for rigorous statistical testing of model fit. It has a number of additional advantages over standard cluster analysis: (a) LCA is not limited to analysis of continuous dependent variables only; (b) LCA can easily accommodate covariates; and (c) LCA is more robust (i.e., has lower misclassification rates) to departures from the assumptions of equal variance and local independence [35].

LCA was conducted with Mplus version 5.1 (Muthén & Muthén) using maximum likelihood estimation. LCA postulates that the association among observed items is due to a discrete latent class structure. The goal of LCA is to identify the smallest number of classes necessary to account for patterns of perceived barriers. The number of latent classes is determined iteratively, beginning with a baseline one-class model and proceeding to test models of increasing numbers of classes. There is currently no gold-standard criterion for determining the optimal number of classes. Therefore, a combination of criteria was used. The statistical criteria used were the Bayesian information criterion (BIC), Lo-Mendell-Rubin's adjusted likelihood ratio test (LRT) [36], entropy measures, and examination of bivariate residuals between pairs of indicators. The BIC is a global measure that balances model fit against model parsimony, with lower BIC values indicating better model fit. The LRT test compares the improvement in fit between adjacent class models (i.e., k-1 class model [null hypothesis] versus k class model [alternative hypothesis]), and provides a p-value that indicates statistically significant improvement in fit. In the current study, α < .05 was considered evidence against the null hypothesis (k-1 classes) in favor of the alternative hypothesis (k classes). Bivariate residuals larger than 1.96 indicated a potential violation of the assumption of local independence in LCA [37].

After determining the optimal number of classes, respondents were assigned to the barrier class that most closely resembled their pattern of perceived barriers (i.e., assigned to the class with the highest posterior class membership probability). The prevalence of respondents in each class is reported, as are the conditional probabilities (probability of reporting a perceived barrier on a specific item conditioned upon class membership). For the purposes of this study, conditional probabilities of 70-100% were considered to be a high probability of a specific barrier, 40-69% was considered a moderate probability, and less than 40% was considered a low probability [38].

To analyze latent class membership and use of health services (aim 2), after assigning respondents to barrier classes, we used multiple logistic regression (STATA version 11.0; College Station, TX) to assess the association between class membership and measures of health service utilization and unmet medical needs. Based on the Andersen model (Figure 1) and prior research on utilization of health services, we hypothesized that respondents in groups perceiving barriers in access would be less likely to receive healthcare services and more likely to have unmet medical need or delayed care.

To analyze factors predicting latent class membership (aim 3), we used multinomial logistic regression to assess how predisposing, enabling, and medical need variables affected the probability of membership in latent barrier classes. Collinearity statistics were used to assess the possible collinearity between covariates. No variance inflation factor exceeded 2.0, suggesting collinearity was not a problem. Multinomial logistic regression makes the assumption known as the independence of irrelevant alternatives (IIA). The Small and Hsiao test of IIA was computed, and the null hypothesis (IIA assumption holds) was not rejected (p > .10). Exponentiated coefficients from multinomial logistic regression yielded relative risk ratios that can be interpreted similarly to odds ratios from logistic regression.

## Results

### Description of Sample

Table 1 illustrates the WLS respondent sample characteristics. The mean age was 64 years, 46% were male, and nearly 80% were married. Eighty-seven percent of respondents had private health insurance, 10% had public insurance only, and 3% reported having no health insurance. The mean number of diagnosed conditions was 3.1, and 15% had depressive symptomatology. The percentage of respondents reporting a barrier in perceived access ranged from 3.7% (prescription services) to 31.9% (out-of-pocket costs).

**Table 1:** Description of Sample.

Study Variables	Mean or Percent
**Predisposing characteristics**	
Age (years), mean (SD)	64.3 (0.7)
Male, %	45.6
Married, %	79.7
Education (years), mean (SD)	13.8 (2.4)
**Individual-level enabling characteristics**	
Total annual household income (USD), mean (SD)	$66,572 ($83,039)
Health insurance	
Any private insurance, %	86.6
Public insurance only, %	10.1
No insurance, %	3.3
**County-level enabling characteristics**	
Large metropolitan, %	33.7
Small metropolitan, %	38.0
Micropolitan, %	15.0
Rural (non-core), %	13.3
**Need characteristics**	
Number of diagnosed conditions, mean (SD)	3.1 (2.3)
Depressive symptomatology, %	15.1
Any ambulation limitations, %	7.3
Any dexterity limitations, %	2.1
Any cognitive limitations, %	25.4
Any pain limitations, %	42.8
Any speech limitations, %	2.0
Any hearing limitations, %	3.9
**Perceived access to health care items**	
...emergency care, %	4.8
...hospital care, %	3.2
...prescription services, %	3.7
...specialty care, %	4.9
...mental health care, %	8.6
...convenience of office location, %	10.3
...advice by phone, %	23.7
...phone appointments, %	5.7
...wait for appointment, %	19.7
...time in waiting room, %	18.8
...time with doctor, %	12.7
...affordability, %	31.9
...doctor is honest, %	2.3
...doctor pays attention, %	3.8
...doctor shares embarrassing info., %	3.6
**Latent class validation items**	
Flu shot... %	62.9
Cholesterol screening... %	78.6
High blood pressure screening... %	93.5
Routine physical exam...%	77.2
Unmet medical need... %	8.5

N = 5,465. Depressive symptomatology was measured using the Center for Epidemiologic Studies - Depression (CES-D) scale. Perceived access items were drawn primarily from the Group Health Association of America's Consumer Satisfaction Survey. Each item begins with the stem, "Thinking about your own health care, how would you rate..." Response categories for perceived access to health care items (Excellent, Very Good, Good, Fair, Poor) were collapsed as follows: 1 = Fair/Poor, 0 = Good, Very Good, Excellent. Use of preventative services measures were used to validate latent class membership and these questions asked if the patient had received the service in the past 12 months or had unmet medical need in the past 12 months.

### Latent Class Analysis (Aim 1 Results)

Models with one to five classes were initially estimated for the 15 perceived barriers in access to health care items. In all models, an extremely high bivariate residual was noted for the two items related to phone services (availability of medical information or advice by phone [advice by phone] and arrangements for making appointments for medical care by phone [phone appointments]). A similarly extreme bivariate residual was also noted for items pertaining to emergency care and hospital care. To address these extreme violations of the local independence assumption, we chose to keep the item in the offending pair that was more prevalent in the sample. Specifically, we retained the item pertaining to information/advice by phone (dropped phone appointments) and the item pertaining to emergency care (dropped hospital care). In addition, the following items were dropped because of their low probability of occurrence in the sample and their inability to assist in distinguishing latent classes: access to prescription services, your doctor is honest with you, your doctor pays attention to you, and your doctor may share embarrassing information [39]. Therefore, all final models were re-estimated using the nine remaining perceived barrier items.

**Figure 2:**
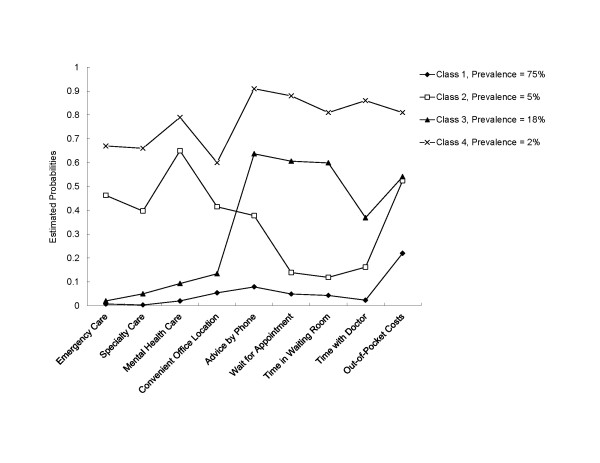
**Predicted probability of reporting a perceived barrier for each access item, conditioned on latent class assignment**.

Table 2 presents the results of the final latent class analysis. According to the Bayesian information criterion (BIC) (lower indicates better fit) and the Lo-Mendell-Rubin adjusted likelihood ratio test (LRT) p-value (4-class > 3-class, p < .001), the 4-class model was superior to models with 3 or fewer classes and the 5-class model. There remained seven bivariate residuals exceeding the z-score of 1.96 in the 4-class model. However, given the relatively high entropy value (0.84) for the 4-class model, as well as the interpretability of the classes, we decided not to model these local dependencies. Accordingly, we adopted the 4-class model. The prevalence of cases in each class and the predicted probability of specific perceived barriers conditioned on latent class assignment are presented in Figure 2.

**Table 2:** (Aim 1 Results).

	Number of Classes				
	
Fit statistic	1	2	3	4	5
Log-likelihood	-17571	-15773	-15450	-15316	-15292
# of estimated parameters	9	19	29	39	49
BIC	35220	31710	31149	30968	31005
P-value (k-1 vs. k)	N/A	P < .001	P < .001	P < .001	P = 0.058 (NS)
Entropy	N/A	0.79	0.82	0.84	0.84
# of bivariate residuals z > 1.96	36	28	15	7	5

Fit Statistics for Latent Class Model of Perceived Barriers in Access to Medical Care Indicating Optimal 4-Class Solution.

For Bayesian information criterion (BIC), a smaller value suggests a better model fit. The likelihood ratio test tests significance in the -2 times Log-likelihood difference between the model with k and k - 1 (H_0_) classes. Entropy values range from zero to one, with values closer to one indicating clearer delineation of latent classes. Reported p-values are for Lo-Mendell-Rubin adjusted log ratio test.

Class 1 contained 75% (n = 4,120) of the respondents. Members of Class 1 had a low probability of perceiving barriers with any attribute of health care access. Given that the defining characteristic of Class 1 relative to other classes is perceiving no major barriers in any dimension of access, we labeled this class "No Barriers Group."

Class 2 contained approximately 5% (n = 248) of the respondents. Overall, members of Class 2 had a higher probability of perceiving barriers in the availability/accessibility of health services. Members of Class 2 had a moderate probability of perceiving barriers in access to mental health care (65%), emergency care (46%), specialty care (40%) and convenience of office locations (42%). Additionally, members of Class 2 had a moderate probability of reporting perceiving affordability barriers from burdensome out-of-pocket costs (52%). Members of Class 2 had a lower probability of perceiving barriers with other attributes of health care access (advice by phone, wait for appointment, time in waiting room, and time with doctor). Given that barriers in availability/accessibility were the defining characteristic of Class 2 relative to other classes, we labeled this class "Availability Barriers Group."

Class 3 contained approximately 18% (n = 1,005) of the respondents. Members of Class 3 had a moderate probability of perceiving problems with length of time waiting between making an appointment for routine care and the day of the visit [wait for appointment] (61%), length of time spent waiting at the office to see the doctor [time in waiting room] (60%), availability of information/advice by phone [advice by phone] (64%), and with out-of-pocket costs [out-of-pocket costs] (54%). Members of Class 3 had a lower probability of perceived problems with the availability/accessibility of health services. The defining characteristic of Class 3 is perceived problems with how well their provider's operation is organized to accommodate their own constraints and preferences. Therefore, we labeled this class "Accommodation Barriers Group."

Class 4 contained approximately 2% (n = 92) of the respondents. Members of Class 4 had a high probability of perceiving problems with access to mental health care (79%), availability of information/advice by phone [advice by phone] (91%), length of time waiting between making an appointment for routine care and the day of the visit [wait for appointment] (88%), length of time spent waiting at the office to see the doctor [time in waiting room] (81%), amount of time with doctors and staff during a visit [time with doctor] (86%), and with out-of-pocket costs (81%). Members of Class 4 also had a moderate probability of perceived problems with access to medical care in an emergency (67%), specialty care (66%), and convenience of office location (60%). The defining characteristic of Class 4 is perceived problems in all represented dimensions of access (recall that perceived barriers pertaining to Acceptability were deleted due to low prevalence and failure to cluster with other classes). Therefore, we labeled this class "Severe Barriers Group."

### Concurrent Validity of Latent Classes (Aim 2 Results)

To examine the concurrent validity of the four latent classes identified in the final LCA model, we examined the association between latent class membership, preventive care use, and potential unmet healthcare needs (Table 3). All results are relative to the "No Barriers Group." Results were consistent with our hypothesis that respondents in barrier groups would be less likely to receive recommended clinical preventive services (flu shot, cholesterol and blood pressure screening, routine physical examination), and more likely to experience unmet medical needs. The strength of the association between barrier class membership and preventive care use/unmet need was particularly high in respondents in the Severe Barriers Group compared to those in the Accommodation or Availability/Accessibility Groups. This finding is consistent with a "dose-response" effect across dimensions of access on preventive care use and potential unmet need because the defining characteristic of individuals in the Severe Barriers Groups was perceived problems with both accommodation and availability/accessibility of services (in addition to affordability).

**Table 3:** Percentage of Older Adults Receiving Clinical Preventive Services and With Unmet Healthcare Needs by Latent Class Membership.

	Latent Class			
	
Health Service/Unmet Need	Class 1 (No Barriers)	Class 2 (Availability)	Class 3 (Accommodation)	Class 4 (Severe Barriers)
Flu shot, %	64.9	57.7*	59.4**	47.7**
Cholesterol screening, %	83.4	75.8**	75.8**	66.5**
Blood pressure screening, %	97.3	95.2*	95.3**	88.9**
Routine physical examination, %	81.3	74.4*	71.5**	63.5**
Unmet medical need, %	5.4	9.2**	11.3**	27.3**

N = 5,465. One asterisk (*) indicates significance at the 5% level while two asterisks (**) indicates significance at the 1% level. Percentages are adjusted for predisposing, enabling, need variables. All statistical significance is relative to the "No Barriers" group.

### Factors that Predict Latent Class Membership (Aim 3 Results)

We used multinomial logistic regression to assess how predisposing, enabling, and medical need variables affected the probability of membership in latent barrier classes (Table 4). All relative risk ratios (RRR) are relative to the likelihood of membership in the No Barriers Group.

**Table 4:** Relative Risk Ratios from Multinomial Logistic Regression Predicting Barrier Class Membership.

	Class 2 vs. Class 1 *Availability Barriers/No Barriers*		Class 3 vs. Class 1 *Accommodation/No Barriers*		Class 4 vs. Class 1 *Severe Barriers/No Barriers*	
			
	RRR	95% CI	RRR	95% CI	RRR	95% CI
**Predisposing characteristics**						
Age in years	0.94	0.78 - 1.14	0.97	0.88 - 1.07	1.17	0.88 - 1.57
Male (reference is female)	1.40*	1.07 - 1.84	1.14	0.99 - 1.31	1.43	0.94 - 2.18
Married (reference is unmarried)	1.06	0.74 - 1.52	0.85	0.70 - 1.04	0.70	0.44 - 1.11
Education in years	1.03	0.98 - 1.09	1.00	0.97 - 1.03	1.02	0.93 - 1.13
**Enabling characteristics**						
Total household income (logged)	1.02	0.98 - 1.06	0.99	0.97 - 1.02	0.96	0.90 - 1.02
Health insurance						
Any private insurance (reference)						
Public insurance only	1.05	0.71 - 1.54	0.86	0.66 - 1.12	1.29	0.68 - 2.44
Uninsured	1.32	0.64 - 2.75	0.89	0.58 - 1.35	3.48**	1.68 - 7.24
County rurality						
Large metropolitan (reference)						
Small metropolitan	1.35	0.94 - 1.05	0.84	0.63 - 1.12	1.23	0.79 - 1.92
Micropolitan	1.63*	1.05 - 2.53	1.02	0.81 - 1.30	1.13	0.64 - 1.99
Rural	4.18**	2.94 - 5.93	0.86	0.66 - 1.13	2.72**	1.62 - 4.57
**Need characteristics**						
Number of diagnosed conditions	1.06*	1.01 - 1.12	1.02	0.98 - 1.06	1.05	0.95 - 1.16
Depressive symptomatology	1.80**	1.32 - 2.44	1.52**	1.26 - 1.86	2.59**	1.58 - 4.26
Any ambulation limitations	1.17	0.75 - 1.86	0.97	0.73 - 1.30	0.91	0.38 - 2.23
Any dexterity limitations	1.20	0.55 - 2.61	1.40	0.85 - 2.29	0.36	0.05 - 2.72
Any cognitive limitations	1.42*	1.08 - 1.86	1.27*	1.07 - 1.49	0.89	0.58 - 1.35
Any pain limitations	1.11	0.82 - 1.49	1.14	0.99 - .131	1.19	0.76 - 1.88
Any speech limitations	1.38	0.66 - 2.86	1.28	0.78 - 2.14	4.09**	1.86 - 9.03
Any hearing limitations	2.18**	1.30 - 3.71	0.94	0.64 - 1.38	1.23	0.27 - 3.22

N = 5,465. One asterisk (*) indicates significance at the 5% level while two asterisks (**) indicates significance at the 1% level. Statistical comparisons are relative to the No Barriers group. RRR stands for Relative Risk Ratio.

The following independent variables were associated with an increased likelihood of being in the Availability/Accessibility Barrier class: being male (RRR: 1.40, 95% Confidence Interval [CI] 1.07 - 1.84, p < .05), living in a micropolitan (RRR: 1.63, 95% CI 1.05 - 2.53, p < .05) or rural county (RRR: 4.18, 95% CI 2.94 - 5.93, p < .01) versus metropolitan area, higher number of diagnosed conditions (RRR: 1.06, 95% CI 1.01 - 1.12, p < .05), depressive symptomatology (RRR: 1.80, 95% CI 1.32 - 2.44, p < .01), cognitive limitations (RRR: 1.42, 95% CI 1.08 - 1.86, p < .05), and hearing limitations (RRR: 2.18, 95% CI 1.30 - 3.71, p < .01).

Only medical need variables were significantly associated with membership in the Accommodation Barrier Group. Specifically, older adults with depressive symptomatology (RRR: 1.52, 95% CI 1.26 - 1.86, p < .01) and cognitive limitations (RRR: 1.27, 95% CI 1.07 - 1.49, p < .05) were more likely to be in the Accommodation Group (versus No Barriers).

The following independent variables were associated with an increased likelihood of being in the Severe Barrier class: having no health insurance coverage versus private insurance (RRR: 3.48, 95% CI 1.68 - 7.24, p < .01), living in a rural county (RRR: 2.72, 95% CI 1.62 - 4.57, p < .01), depressive symptomatology (RRR: 2.59, 95% CI 1.58 - 4.26, p < .01), and speech limitations (RRR: 4.09, 95% CI 1.86 - 9.03, p < .01).

## Discussion

To the best of our knowledge, this is the first study to use latent class analysis (LCA) to identify a typology of perceived barriers to healthcare in older adults across multiple dimensions of access. For older adults in the community, many of whom have complex healthcare needs requiring ongoing contact with a range of healthcare provider types, our results suggest the existence of four distinct classes of individuals who differ significantly with respect to their patterns of perceived access barriers. Furthermore, we found that older adults in any of the classes comprised of individuals with perceived barriers were less likely to receive recommended preventive care, and more likely to report unmet medical needs. The identified latent class structure, therefore, may have important implications for identifying subgroups of older adults whose compromised ability to access timely and effective medical care may increase their risk of developing preventable diseases [2] complications from unmanaged illnesses [5] and serious adverse health outcomes [40].

Results from the LCA models revealed that 75% of older adults had a low probability of perceiving any barriers, while the other 25% clustered into one of three barriers groups. Specifically, 5% of older adults perceived barriers pertaining to the availability of healthcare providers. Another 18% of older adults reported barriers with how well healthcare providers' offices are organized in ways that accommodate their own constraints and preferences (for example, how soon they can get in to be seen, amount of time with providers). The final 2% of older adults perceived barriers across all four dimensions of access. Results also revealed that healthcare affordability issues are a problem shared by members of all three barrier groups, suggesting that the 25% of older adults are highly likely to perceive barriers in multiple access domains. On the one hand, this identified latent class structure suggests that reducing financial burden of healthcare services (for example, Medicare Part D prescription drug plan) is necessary for improving access to care in older adults. On the other hand, our findings also suggest that policies focusing exclusively on affordability barriers may be insufficient for achieving these goals because all members of these barrier groups perceived co-occurring barriers in other dimensions of access.

Overall, predisposing factors were not strongly related to perceived barrier groups. However, members of the Availability/Accessibility Barriers group were more likely to be men than women, even after adjusting for differences in other predisposing, enabling, and need variables. Previous research in older adults suggests that women use more primary care services and are more likely to receive preventive care compared to men. Because men do not use primary care as often as women, they may be less familiar with the range of healthcare services available to them. Alternatively, elder men may be more sensitive to travel times and, therefore, less willing or able to travel the same distance as women to obtain care [38]. Future research should explore the underlying reasons why older men may perceive problems with the availability of services.

Older adults without health insurance were over three times more likely to be in the Severe Barriers Groups. Further analysis reveals that over 90% of the uninsured in this sample (consisting of adults aged 63-67) were under age 65 and, therefore, likely ineligible for Medicare insurance benefits. Because these group members perceived co-occurring barriers in all dimensions of access, policies designed to expand insurance coverage to reach the uninsured near elderly may be particularly beneficial for improving access in this vulnerable subgroup of older adults [41]. We also found that members of both the Severe Barrier and Availability/Accessibility Barrier Groups were more likely to be residing in rural counties. The delivery of health services to rural communities is a long-standing challenge to policy makers, and a substantial literature documents the potential barriers to primary and specialty care among older rural populations [42,43]. In addition to geographic barriers to services, a subset of older adults in rural communities perceive a constellation of access barriers. These results further underscore the range in types of barriers that many older adults must overcome to access medical care, and the need for multi-pronged solutions to address the different barrier types.

This study has implications for the provision of healthcare services for older adults with chronic conditions and those with functional disabilities. Specifically, we found that older adults in all three barriers groups appeared to have the greatest potential need for healthcare services. We found that older adults with symptoms of depression, for example, were more likely to be classified into all three barriers groups. Further, those with cognitive limitations were more likely to report problems with Availability/Accessibility and Accommodation barriers, and those with speech difficulties were over four times more likely to report barriers in all dimensions of access. These findings are consistent with prior research on the negative impact of depression and functional disability on access to services [12,44,45]. The current 15-minute primary care office visit model is known to be ill-suited for patients with complex health care needs [46]. Alternative models of care, such as the patient-centered medical home [47], have the potential to improve access to care for this older adult population. Numerous statewide demonstrations are underway to examine the medical home's efficacy [48].

Several study limitations should be noted. First, the WLS includes a cohort of largely non-minority graduates from Wisconsin high schools in 1957, which may limit generalizability to cohorts from different years, racial-ethnic minorities, those who did not complete high school, and older adults outside of Wisconsin. Second, because of this study was cross-sectional and non-experimental, we cannot confirm that the identified associations are causal. Third, although we included in our latent class analysis a range of access items representing multiple dimensions of access, the WLS items for measuring perceived access were not designed to evaluate the full complexity of access dimensions described by Penchansky; as a result, there was insufficient item "coverage" to establish discriminant validity between Availability and Accessibility dimensions. And finally, utilization of services was assessed via respondent self-report, and recall bias is a potential concern.

## Conclusions

Despite these limitations, the application of latent class analysis to develop a typology of perceived barriers in access has implications for future research and policy. LCA identified three meaningful subgroups of perceived barrier types. In this study, none of these three barrier subgroups perceived only one type of barrier; rather, older adults with perceived barriers tended to report multiple, co-occurring problems with access. Multi-pronged interventions and policy adjustments may, therefore, be specifically targeted to address the needs of discrete clusters of individuals facing similar sets of barriers. The risk factors identified in the current study could potentially assist healthcare organizations and providers with targeting polices and interventions designed to improve access in their most vulnerable older adult populations, particularly those in rural areas, with functional disabilities, or in poor mental health.

## Competing interests

The authors declare that they have no competing interests.

## Authors' contributions

JMT conceptualized the study, conducted the data analysis, interpreted results, and drafted the manuscript. CTT conceptualized the study, interpreted results, and drafted the manuscript. KAK and NP drafted the manuscript. All authors read and approved the final manuscript.

## Pre-publication history

The pre-publication history for this paper can be accessed here:

http://www.biomedcentral.com/1472-6963/11/181/prepub
